# Evaluation of the role of unconventional prefoldin RPB5 interactor (URI1) in hepatitis B virus infection

**DOI:** 10.1186/s12985-024-02617-2

**Published:** 2025-01-10

**Authors:** Karolína Štaflová, Aleš Zábranský, Iva Pichová

**Affiliations:** Institute of Organic Chemistry and Biochemistry, Czech Academy of Sciences, Prague, Czech Republic

**Keywords:** Unconventional prefoldin RPB5 interactor, URI1, Hepatitis B virus, HBV, Hepatocellular carcinoma

## Abstract

Hepatitis B virus (HBV) infection can cause liver disease and lead to hepatocellular carcinoma (HCC). To better understand the factors involved in viral infection and pathogenesis and to develop novel therapies, it is crucial to investigate virus–host interactions. HBV infection has been shown to increase the expression of the unconventional prefoldin RPB5 interactor (URI1), a cellular protein that promotes liver tumorigenesis and HCC metastasis. Our study investigated the role of URI1 in HBV infection in vitro. Although previous reports have suggested that URI1 may act as an HBV restriction factor, our results showed that URI1 silencing or overexpression did not affect HBV replication in HepG2-NTCP cells. In primary human hepatocytes, URI1 knockdown modestly reduced HBV markers but did not significantly alter acute infection. Supporting the premise that URI1 is a promising therapeutic target for HCC, our findings show that URI1 knockdown does not enhance HBV infection in an acute infection model. This suggests that URI1 may be a viable therapeutic target for patients with HBV-associated HCC without increasing HBV-related complications.

**Supplementary Information:**

The online version contains supplementary material available at 10.1186/s12985-024-02617-2.

Hepatitis B virus (HBV) is a human pathogen that causes acute and chronic liver disease. Currently, more than 250 million people worldwide suffer from chronic HBV infection. These individuals face an elevated risk of liver cirrhosis and cancer. Chronic HBV infection promotes the development of liver cancer by causing chronic inflammation, activating oncogenic signaling pathways or by the integration of viral DNA into the host genome. While existing HBV therapies can effectively control viral replication, they rarely lead to a complete cure resulting in elimination of the viral reservoir [1]. To develop novel effective therapies for HBV infection and associated liver disease, it is crucial to understand the interactions between the virus and the host cell.

Unconventional prefoldin RPB5 interactor (URI1) plays a role in the regulation of cellular metabolism [2, 3]. High expression of URI1, known to be involved in the development of hepatocellular carcinoma (HCC), is associated with poor HCC prognosis [3, 4]. URI1 inhibits NAD + synthesis and promotes hepatocarcinogenesis by inducing DNA damage [3, 5]. Additionally, URI1 expression promotes cancer cell survival [2] and HCC metastasis [6, 7]. Overexpression of URI1 in liver tumor tissue can also promote resistance to cancer therapy [8, 9].

Notably, high URI1 expression has been specifically linked to hepatitis B virus: URI1 levels in liver tumor tissue have been shown to positively correlate with HBV infection, while overexpression of the viral protein HBx has been shown to increase URI1 expression in cell cultures [3, 4]. URI1 has also been implicated in HBV-associated pathogenesis. It has been demonstrated that the co-expression of URI1 and HBx cooperatively promotes tumor growth in mice [10].

Because of its role in liver tumorigenesis, URI1 has been proposed as a target in HCC therapy [6, 11]. Notably, downregulating URI1 expression during the early stages of liver tumorigenesis was shown to have therapeutic effects in a mouse HCC model [3]. Additionally, a recent study demonstrated how URI1-mediated cancer treatment resistance can be overcome in vitro. URI1 overexpression promotes resistance, via stearoyl-CoA desaturase 1 (SCD1), to ferroptosis induced by tyrosine kinase inhibitors. URI1 knockdown or inhibition of SCD1 has been shown to enhance hepatocyte sensitivity to tyrosine kinase inhibitors [8].

URI1 also shows promise as a potential therapeutic target in HBV-associated HCC. However, it is important to understand the possible interplay between URI1 and the viral infectious cycle. Previous studies suggest that URI1 may act as a restriction factor of HBV replication. For instance, URI1 overexpression was shown to suppress HBx-driven transcription from a plasmid with an HBV transcription enhancer [12], while overexpression of URI1 reduced HBV replication in mice injected with an HBV-encoding plasmid [13]. However, the effect of URI1 on HBV replication has not been investigated using an infectious virus.

The aim of our study was to investigate the effect of URI1 on HBV infection in vitro. Contrary to previous studies, which suggest that URI1 may be a potential restriction factor for HBV infection, we found that URI1 level did not affect HBV replication either in HepG2-NTCP cells or in primary human hepatocytes (PHH). We propose that this discrepancy may be attributed to differences in the experimental models used to study HBV replication.

To study the effect of URI1 on HBV infection in vitro, we chose two hepatocyte models. The HepG2 hepatoblastoma cell line has been previously used to study URI1 function and its interaction with HBV [3, 4, 10]. HepG2-NTCP cells express the sodium taurocholate co-transporting polypeptide (NTCP) receptor, rendering these cells susceptible to HBV infection. While this cell line is a common model used in HBV research, it does not fully replicate physiological hepatic functions [14, 15]. Currently, primary human hepatocytes are considered the most relevant cell culture model for studying HBV infection [14]. Therefore, we also analyzed the effect of URI1 on HBV infection in primary human hepatocytes.

First, we decided to test the effect of URI1 knockdown on HBV infection. We tested URI1 silencing using three commercially available siRNAs (URI1 siRNA 1–3, Supplementary Table 1) and compared their efficiencies in the HepG2-NTCP cell line and PHH. Cells were transfected with either a control or URI1-targeting siRNA using Lipofectamine RNAiMAX (Invitrogen). Two days after transfection, URI1 knockdown was evaluated using RT-qPCR and western blotting. All tested URI1 siRNAs efficiently decreased URI1 mRNA levels by more than 70% (Fig. S1A, Fig. S1B). Silencing was not toxic in either HepG2-NTCP cells or PHH, as documented by the XTT cell proliferation assay (Roche, #11465015001) in HepG2-NTCP cells (Fig. S1C) and by the CellTiter-Glo assay (Promega) in PHH (Fig. S1D).

To determine the effect of URI1 silencing on HBV infection of HepG2-NTCP cells and PHH, we performed URI1 siRNA knockdown using Lipofectamine RNAiMAX (Invitrogen) in both cell types. The following day, HepG2-NTCP cells were infected with HBV (genotype D) at a multiplicity of infection (MOI) of 500 viral genome equivalents (VGE) per cell in the presence of 4% PEG 8000 and 2% DMSO. After 16 h, the cells were washed three times with PBS and maintained in Dulbecco’s modified Eagle’s medium supplemented with 3% FBS and 2% DMSO. The HBV virus used for infection was prepared by PEG 8000 precipitation, as previously described [16]. Primary human hepatocytes (BioIVT, #M00995-P, lot SRT) were maintained in Williams’ E medium supplemented with primary hepatocyte maintenance supplements (Gibco), 2% FBS, and 1.4% DMSO, with medium changes every three days. One day after URI1 silencing, PHH were infected with HBV at an MOI of 300 VGE per cell. We analyzed the markers of viral replication five days after infection for HepG2-NTCP cells and seven days after infection for PHH (for the experimental timeline, see Fig. 1A). To confirm efficient viral replication in our infection models, we quantified viral antigen secretion using a chemiluminescent immunoassay (CLIA), which confirmed active HBV replication in both cell types (Fig. S2A). Additionally, immunofluorescence staining of the HBc protein allowed us to quantify infected hepatocytes, with an estimated infection efficiency of approximately 20% for HepG2-NTCP cells and 40% for PHH (Fig. S2B).

**Fig. 1:**
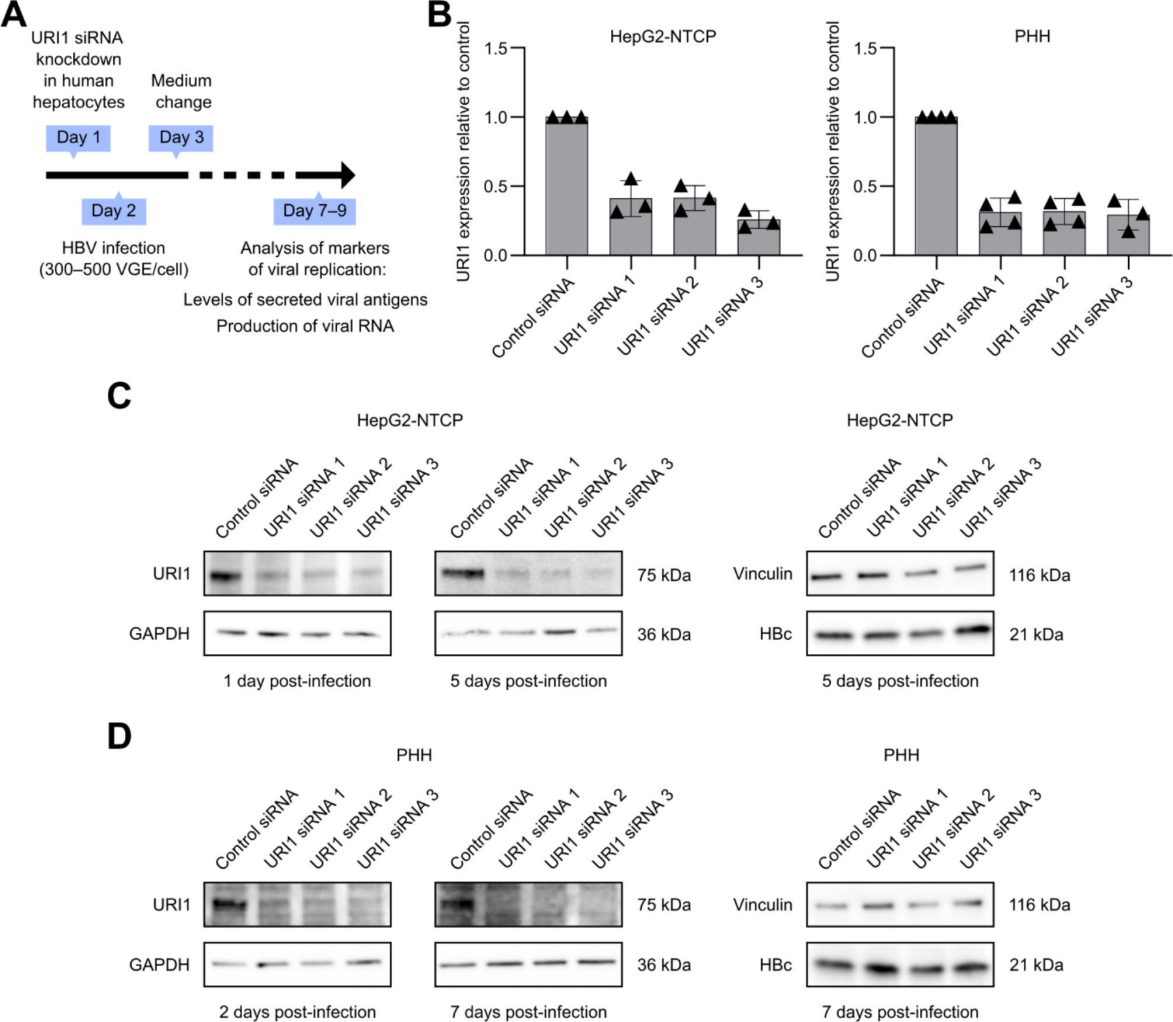
URI1 silencing in HBV-infected human hepatocytes. **(A)** Experimental timeline. **(B)** URI1 mRNA expression in HBV-infected cells was determined by RT-qPCR 5 days post-infection in HepG2-NTCP cells and 7 days post-infection in PHH. Data represent the mean ± SD; triangles indicate the means of independent experiments. **(C)** URI1 protein expression in HepG2-NTCP cells was analyzed by western blot 1 day and 5 days post-infection. HBc protein expression was analyzed 5 days post-infection. **(D)** URI1 protein expression in PHH was analyzed by western blot 2 days and 7 days post-infection. HBc protein expression was analyzed 7 days post-infection

To ensure efficient URI1 silencing throughout the infection time course, we quantified URI1 mRNA levels using RT-qPCR and protein levels using western blotting. Previous studies have associated HBV infection with increased expression of URI1 [3, 4], highlighting the importance of verifying URI1 knockdown in our experimental setup. RT-qPCR was done using the Luna Universal One-Step RT-qPCR Kit (New England Biolabs) on a CFX Opus 96 Real-Time PCR System (Bio-Rad). Primer pairs for URI1 mRNA quantification and normalization to GAPDH expression are listed in Supplementary Table 2. Five days post-infection, URI1 mRNA levels in HepG2-NTCP cells remained reduced by 40 to 70% compared to the control, while all siRNAs tested reduced URI1 mRNA levels in PHH by approximately 70% seven days post-infection (Fig. 1B). URI1 knockdown efficiency was also confirmed by western blotting. URI1 level was analyzed one day and five days post-infection in HepG2-NTCP cells, and two and seven days post-infection in primary human hepatocytes. Cell lysates were prepared using the CelLytic M buffer (Sigma-Aldrich), and 20 µg of lysate was separated by SDS-PAGE on 4–15% Mini-PROTEAN TGX precast protein gels (Bio-Rad). After protein transfer onto a nitrocellulose membrane, URI1 was detected using a specific primary antibody (Sigma-Aldrich, #HPA071709) and an HRP-conjugated secondary antibody (Sigma-Aldrich, #AP307P). Equal protein loading was confirmed by detecting GAPDH (Invitrogen, #MA5-15738; Sigma-Aldrich, #A4416) on the same membrane. Western blot analysis confirmed that all the tested siRNAs efficiently downregulated URI1 levels in HBV-infected cells (Fig. 1C and D). Following the URI1 silencing, the viral replication in infected hepatocytes was confirmed by the detection of HBc protein (Fig. 1C and D). For the detection of HBc, 10 µg of lysate was separated by SDS-PAGE on 4–20% Mini-PROTEAN TGX precast protein gels (Bio-Rad). Vinculin (Sigma-Aldrich, #V9131) was detected on the same membrane as HBc (Gilead Sciences, rabbit monoclonal antibody #53) to confirm equal protein loading.

To analyze how URI1 silencing affected HBV virus infection, we measured extracellular levels of the viral antigens HBsAg and HBeAg from cell supernatants using a commercial ELISA kit (Bioneovan). After URI1 silencing in HepG2-NTCP cells, we observed no significant changes in HBsAg secretion compared to the control (Fig. 2A). However, in PHH, we observed a significant decrease in the extracellular level of HBsAg for two of the three URI1-targeting siRNAs used (Fig. 2A). For the remaining siRNA (URI1 siRNA 1), we detected a 20% decrease, which did not reach statistical significance. In contrast to HBsAg secretion, we did not detect any effect of URI1 silencing on HBeAg levels in either cell type (Fig. 2B).

**Fig. 2:**
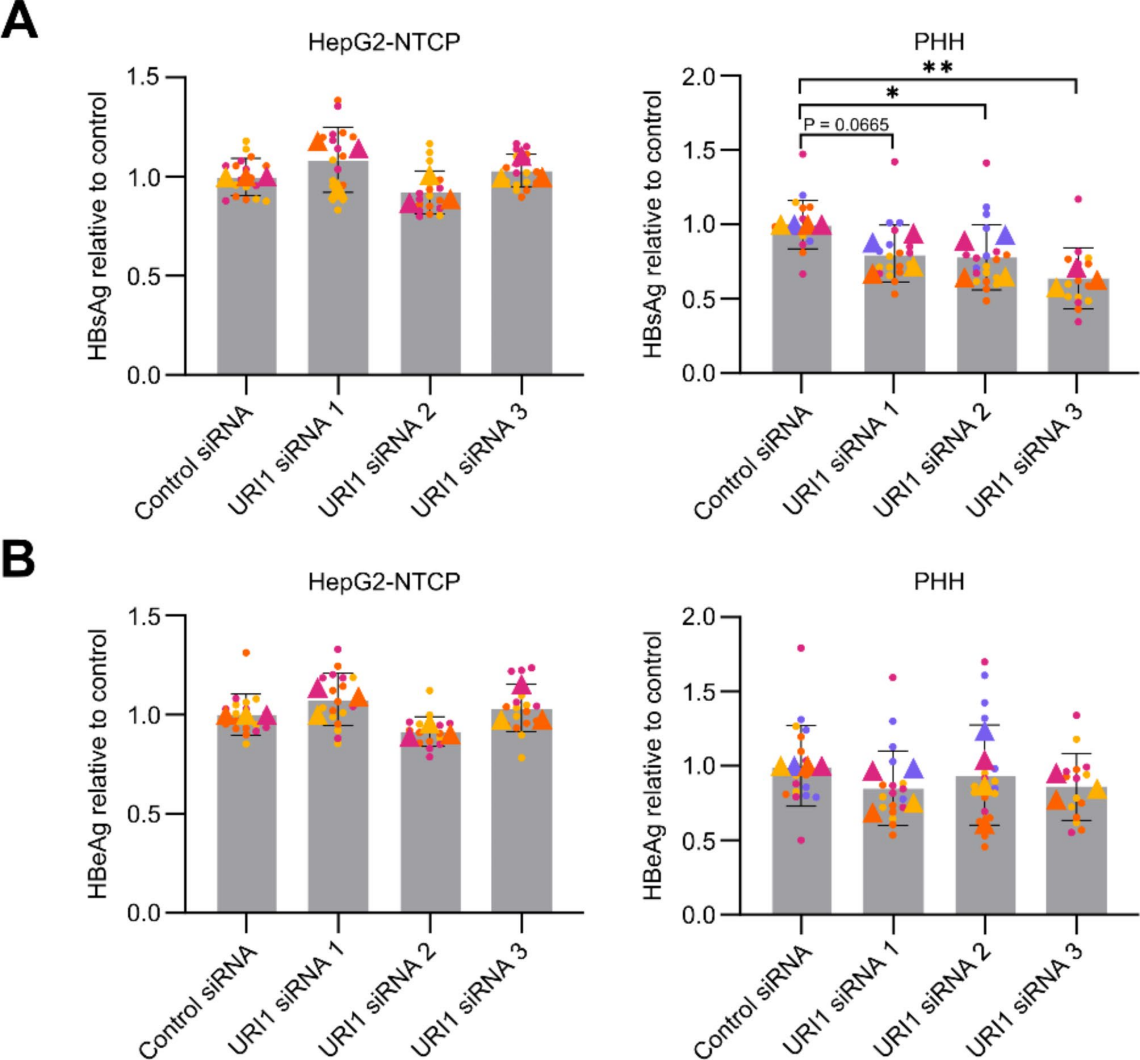
Effect of URI1 silencing on HBV antigen secretion. **(A-B)** Viral antigen secretion was evaluated 5 days post-infection in HepG2-NTCP cells and 7 days post-infection in PHH. Data represent the mean ± SD of at least three experiments; replicates from independent experiments are differentiated by color, with triangles indicating the means of independent experiments. **(A)** HBsAg and **(B)** HBeAg levels in the culture medium were measured by ELISA. Statistical significance of differences between URI1 silencing groups and control treatments was evaluated by ANOVA (* *P* < 0.05, ** *P* < 0.01)

We further investigated the effect of URI1 knockdown on HBV RNA production. Total cell RNA was isolated from HepG2-NTCP cells using the RNeasy Mini Kit (Qiagen), and complete removal of DNA was performed using DNase I treatment. RT-qPCR was performed using the Luna Universal One-Step RT-qPCR kit (New England Biolabs). Primer pairs used for quantifying total viral RNA (HBV-F, HBV-R) and HBV 3.5 kb RNA (pg-F, pg-R) and normalizing to GAPDH expression are listed in Supplementary Table 2. Similar to the detection of viral antigens, URI1 silencing did not affect the production of total HBV RNA (Fig. 3A) or HBV 3.5 kb RNA (Fig. 3B) in HepG2-NTCP cells.

The production of viral RNAs in HBV-infected PHH was assessed by RT-qPCR after isolation of total RNA using the Direct-zol-96 RNA kit (Zymo Research). Again, we analyzed the production of total HBV RNA (Fig. 3A) and HBV 3.5 kb RNA (Fig. 3B). Compared to the control, we observed a decrease in mean HBV RNA production after URI1 silencing. However, this decrease ranged only from 15 to 30% between different siRNAs and was statistically significant only for knockdown with URI1 siRNA 1. This observation is similar to the measurement of secreted HBsAg in PHH (Fig. 2A), where a decrease in mean HBsAg levels was observed, but the difference was not statistically significant for all tested siRNAs.

**Fig. 3:**
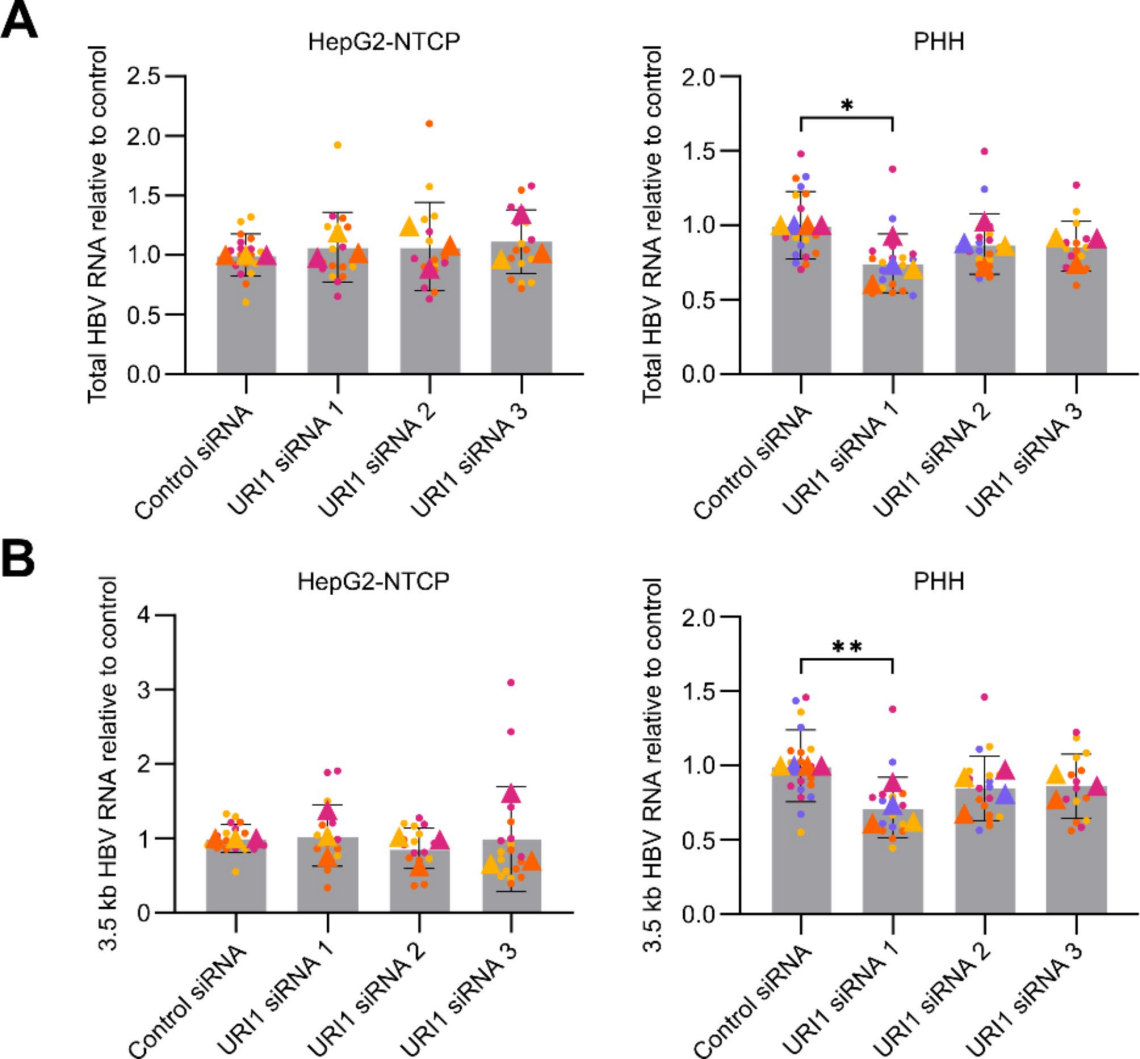
Effect of URI1 silencing on HBV RNA production. **(A)** Total HBV RNA and **(B)** HBV 3.5 kb RNA expression was determined by RT-qPCR 5 days post-infection in HepG2-NTCP cells and 7 days post-infection in PHH. Data represent the mean ± SD from at least three independent experiments; replicates from independent experiments are differentiated by color, with triangles indicating the means of independent experiments. Statistical significance between the URI1 silencing groups and control treatments was assessed by ANOVA (* *P* < 0.05, ** *P* < 0.01)

Since previous studies indicated that HBV replication might be specifically restricted by URI1 overexpression, we also tested the effect of URI1 overexpression on HBV infection in HepG2-NTCP cells. In an earlier report, overexpression of URI1 in the HepG2 cell line was shown to inhibit HBx-driven transcription from a reporter plasmid controlled by HBV enhancer I elements [12].

We transfected HepG2-NTCP cells with either a control plasmid or a URI1-encoding plasmid using Lipofectamine 3000 Reagent (Invitrogen) according to the manufacturer’s instructions. The pcDNA4/V5-His A plasmid (Life Technologies) was used as a control vector. For URI1 overexpression, we used the pcDNA4 URI1 and pCMV5 HA-URI (MRC PUU, #DU31915) plasmids, the latter encoding an N-terminal HA-tagged URI1. To construct the pcDNA4 URI1 plasmid, we amplified the URI1 sequence from pCMV5 HA-URI1 using the primers 10-F and 10-R (see Supplementary Table 2). We then cloned the URI1 insert into the pcDNA4/V5-His A plasmid via the HindIII and NotI restriction sites. Overexpression of URI1 or HA-URI1 was confirmed to be non-toxic to cells, as indicated by XTT proliferation assays conducted at 2, 4, and 7 days after transfection (Fig. S3).

One day after transfection, HepG2-NTCP cells were infected with HBV at an MOI of 500 VGE per cell (Fig. 4A). URI1 overexpression was confirmed at the mRNA level by RT-qPCR on day 6 (Fig. 4B) and at the protein level by western blotting on days 1 and 6 after infection (Fig. 4C). The expression of viral protein HBc was detected 6 days after infection (Fig. 4C). Notably, HA-URI1 overexpression was more efficient than untagged URI1 at both the protein and mRNA levels. Six days after infection, we analyzed markers of viral replication. Following URI1 overexpression, the secretion of viral antigens HBsAg and HBeAg (as measured by CLIA assay, Autobio Diagnostics) remained unchanged (Fig. 4D). Similarly, the URI1 expression level did not affect the production of viral RNAs (Fig. 4E).

**Fig. 4:**
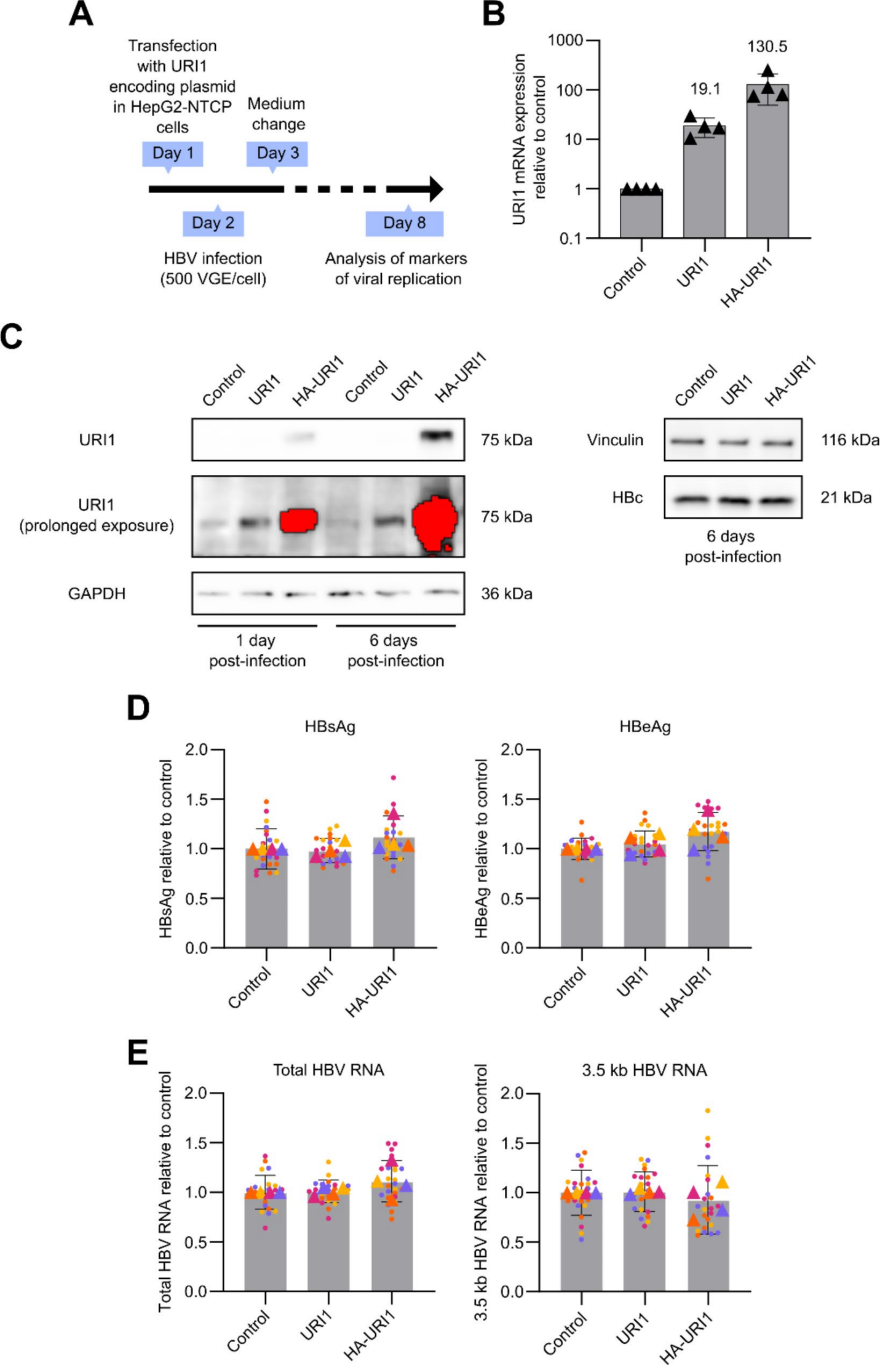
Effect of URI1 overexpression on HBV infection in HepG2-NTCP cells. **(A)** Experimental timeline. **(B)** URI1 mRNA expression following overexpression was determined by RT-qPCR 6 days after infection. Data represent the mean ± SD; triangles indicate the means of independent experiments. Data are shown on a logarithmic scale. **(C)** URI1 and HA-URI1 protein levels were analyzed by western blotting 1 and 6 days after infection. HBc protein level was detected 6 days after infection. Equal protein loading was confirmed by detecting GAPDH or vinculin on the same membrane. Saturated pixels are highlighted in red. **(D-E)** Viral antigen secretion and RNA production were evaluated 6 days after infection. Data represent the mean ± SD of four independent experiments; replicates from independent experiments are differentiated by color, with triangles indicating the means of independent experiments. **(D)** HBsAg and HBeAg levels in the culture medium were measured by CLIA assay. **(E)** Total HBV RNA and HBV 3.5 kb RNA expression were determined by RT-qPCR

Overall, we did not observe an effect of URI1 silencing or overexpression on HBV replication in HepG2-NTCP cells. Our results suggest that URI1 knockdown has a modest negative effect on HBV replication in PHH. However, this effect was not observed for all tested markers of viral replication, and we did not detect an effect of URI1 silencing on HBeAg secretion. Furthermore, the absence of statistically significant differences in HBsAg secretion and HBV RNA production across all siRNAs suggests that URI1 does not play a crucial role in acute HBV infection.

Our observations differ from previous studies, which suggest that URI1 may restrict HBV infection [12, 13]. This discrepancy could be attributed to differences in the experimental models used to study HBV replication. Previous studies have examined the effect of URI1 on HBV expression from a plasmid, whereas our study focused on viral replication following in vitro infection of human hepatocytes. Note that HBV expression from a plasmid differs from that of viral covalently closed circular DNA (cccDNA), which forms a minichromosome complex with cellular proteins, including histones. Consequently, cccDNA regulation is intricately linked to epigenetic modifications and histone organization [17, 18]. Additionally, plasmid transfection omits crucial steps of the viral infection process, such as viral entry and uncoating.

Previous studies have also used systems with overexpressed HBx protein [12, 13], which results in HBx levels exceeding those typically observed in HBV infection, altering the cellular localization of the protein [19]. Elevated HBx levels may also modulate cellular signaling pathways and interactions differently from the controlled expression seen in infected cells [20], potentially affecting study outcomes and interpretations.

Understanding the influence of URI1 on HBV replication may prove important, given the potential of URI1 as a therapeutic target in the treatment of HCC. Targeting URI1 could potentially affect HBV replication, thereby increasing the risk of HBV reactivation and exacerbating liver injury in HBV-positive patients. Our results, which show that URI1 knockdown does not promote HBV infection in an acute infection model, suggest that URI1 may be a promising therapeutic target for HBV-associated HCC patients. However, it should be noted that our study focused on acute HBV infection only. Future studies will need to investigate the effect of URI1 in chronic HBV infection.

## Electronic supplementary material

Below is the link to the electronic supplementary material.

Supplementary Material 1

## Data Availability

Data will be made available on request.

## Abbreviations

cccDNA: Covalently closed circular DNA
CLIA: Chemiluminescent immunoassay
HBV: Hepatitis B virus
HCC: Hepatocellular carcinoma
MOI: Multiplicity of infection
NTCP: Sodium taurocholate co-transporting polypeptide
PHH: Primary human hepatocytes
SCD1: Stearoyl-CoA desaturase 1
URI1: Unconventional prefoldin RPB5 interactor
VGE: Viral genome equivalents
