# Supplementary material for: Evaluation of the role of unconventional prefoldin RPB5 interactor (URI1) in hepatitis B virus infection
Source: Virol J. 2025 Jan 10;22:7. doi: 10.1186/s12985-024-02617-2 (PMC11721529; doi:10.1186/s12985-024-02617-2)
Supplement: Supplementary file 1 — Supplementary Material 1 [file 12985_2024_2617_MOESM1_ESM.docx]

**Supplementary materials**

**Evaluation of the role of unconventional prefoldin RPB5 interactor (URI1) in hepatitis B virus infection**

Karolína Štaflová^1^, Aleš Zábranský^1^, Iva Pichová^1*^

^1^ Institute of Organic Chemistry and Biochemistry of the Czech Academy of Sciences, Prague, Czech Republic

* Correspondence:

Iva Pichová

iva.pichova@uochb.cas.cz

**Supplementary Table 1** List of siRNAs used

| **siRNA** | **Manufacturer information** |
| --- | --- |
| Control siRNA | Merck, #SIC001 |
| URI1 siRNA 1 | Thermo Fisher, #s16621 |
| URI1 siRNA 2 | Thermo Fisher, #s16623 |
| URI1 siRNA 3 | Origene, #SR322507A |

**Supplementary Table 2** List of used primers

| **Primer** | **Primer sequence (5′ to 3′)** |
| --- | --- |
| URI1-F | AGAATCCAGCATTGGAAGAAGG |
| URI1-R | CTGGCATGAAGGCAAAAGGG |
| GAPDH-F | GAAGGTCGGAGTCAACGGATTT |
| GAPDH-R | CGTTCTCAGCCTTGACGGT |
| HBV-F | AGAGGACTCTTGGACTCTCAGC |
| HBV-R | CCTCCCAGTCTTTAAACAAACAGTC |
| pg-F | CACCTCTGCCTAATCATC |
| pg-R | GGAAAGAAGTCAGAAGGCAA |
| 10-F | TATATAAAGCTTCACCATGGAGGCGCCCACCGTG |
| 10-R | TATATAGCGGCCGCTAGTCTTTCTGTT |


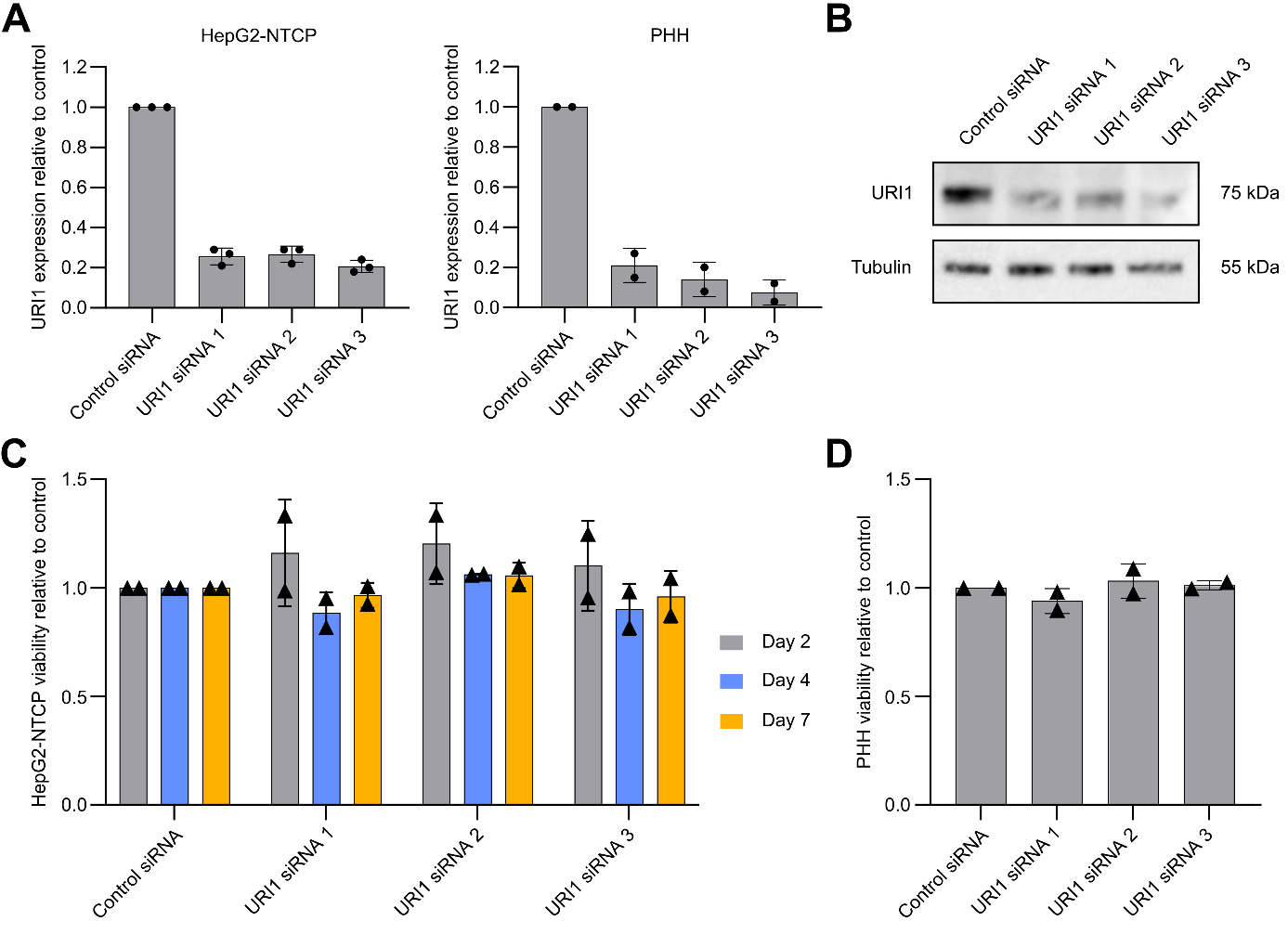


**Fig. S1** URI1 silencing in HepG2-NTCP cells and PHH. (**A**) URI1 expression 2 days after silencing was determined by RT-qPCR. Total cell RNA was isolated using the Direct-zol RNA Microprep kit (Zymo Research) and RT-qPCR was performed using the Luna Universal One-Step RT-qPCR Kit (New England Biolabs). Primer pairs used for quantifying URI1 mRNA and for normalization to GAPDH expression are listed in Supplementary Table 2. All tested URI1 siRNAs efficiently decreased URI1 expression by approximately 70% in HepG2-NTCP cells and 80% in PHH. Points represent measurements from independent experiments. Data represent mean ± SD. (**B**) URI1 expression in HepG2-NTCP cells was analyzed by western blot 2 days after silencing. URI1 was detected with a specific primary antibody (Sigma-Aldrich, #HPA071709) and an HRP-conjugated secondary antibody (Sigma-Aldrich, #AP307P). Equal protein loading was confirmed by detecting tubulin (Invitrogen, #62204; Sigma-Aldrich, #A4416) on the same membrane. (**C**) Cell viability after URI1 silencing in HepG2-NTCP cells was determined by XTT assay at 2, 4, and 7 days after URI1 knockdown. Data represent mean ± SD of two independent experiments. Means from independent experiments are presented as triangles. (**D**) Cell viability eight days after URI1 silencing in PHH was determined by CellTiter-Glo assay. Data represent mean ± SD of two independent experiments. Triangles represent means from independent experiments.


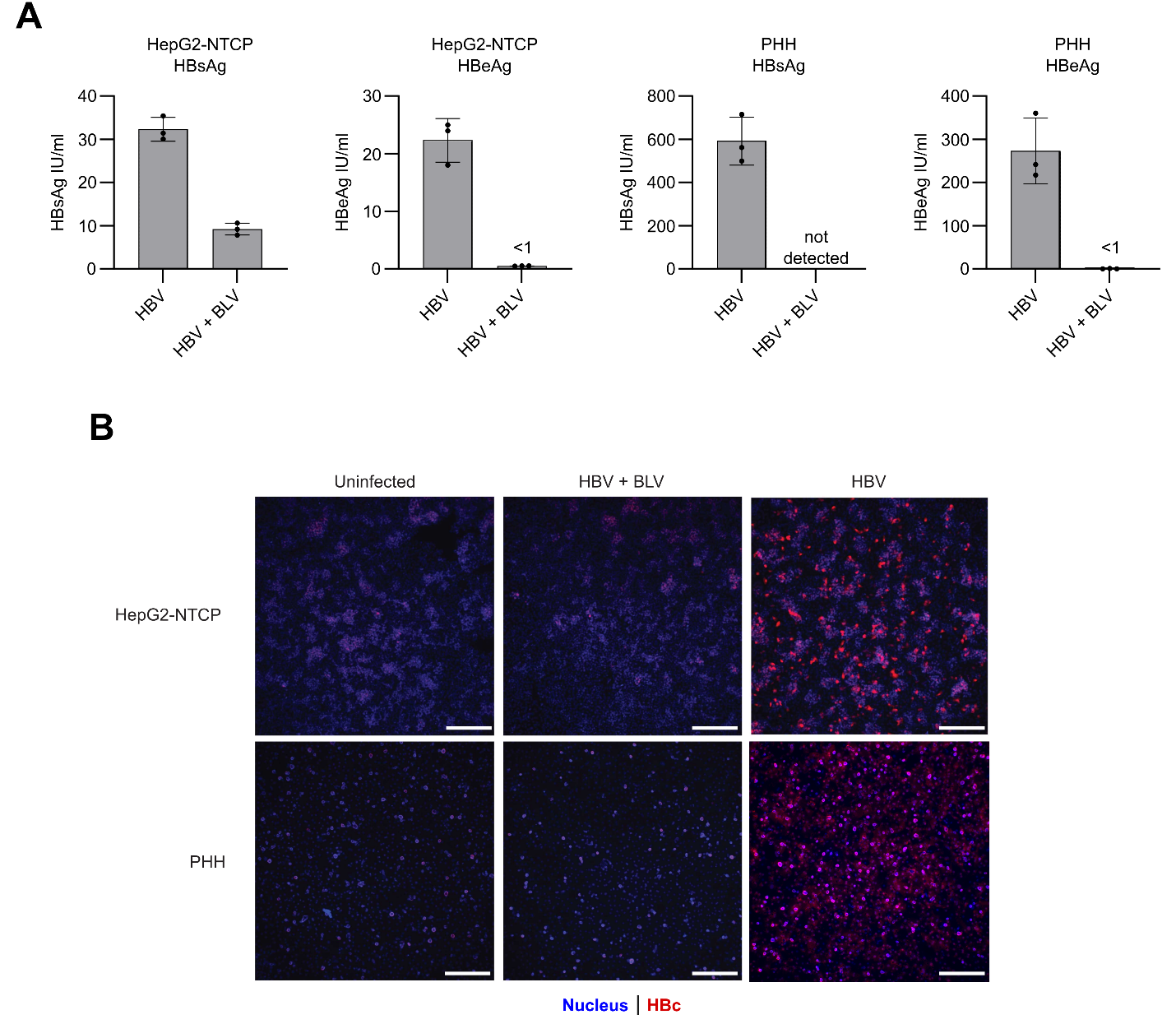


**Fig. S2** HBV infection in HepG2-NTCP cells and PHH. (**A-B**) HepG2-NTCP cells were infected with HBV at an MOI of 500 VGE/cell, while PHH were infected at an MOI of 300 VGE/cell. As a control, cells were pretreated with the HBV entry inhibitor bulevirtide (BLV; Seleckchem, 500 nM) for three hours prior to infection; the inhibitor was also present during incubation with the viral inoculum. Viral infection was analyzed five days after infection for HepG2-NTCP cells and seven days after infection for PHH. **(A)** HBsAg and HBeAg secretion were quantified by CLIA assay (Autobio Diagnostics). Data are presented as the mean ± SD. Points represent replicates. (**B**) HBV-infected hepatocytes were visualized by immunofluorescence staining of the viral protein HBc. Cells were washed with PBS, fixed with 4% paraformaldehyde in PBS for 15 minutes at room temperature, then washed with PBS and permeabilized with 0.2% Triton X-100 in PBS for 5 minutes. HBc was detected using a specific primary antibody (Gilead Sciences, #53) and a Cy3-conjugated secondary antibody (Jackson ImmunoResearch, #711-007-003). Images were acquired using an Olympus IX81 microscope and infection efficiency was determined using ScanR Analysis 3.4.1 software (National Instruments Corporation) by comparing the total number of DAPI-stained nuclei to the number of cells stained with anti-HBc antibody. The HBV infection efficiency was approximately 40% for PHH and 20% for HepG2-NTCP cells, with background levels of around 3%, which were comparable between uninfected and HBV-infected BLV-treated cells in both cell culture models. The scale bar represents 300 µm.

**
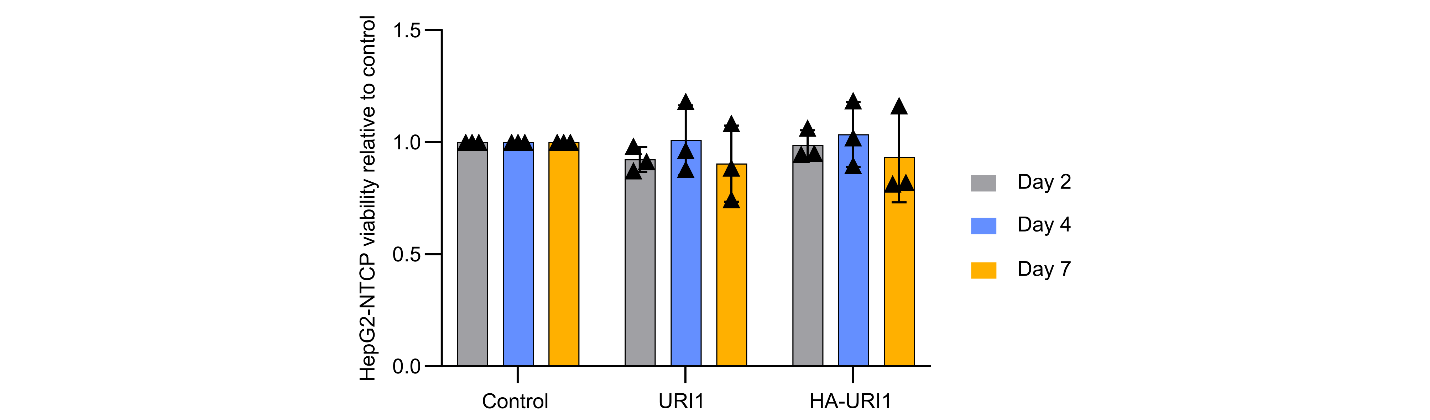
**

**Fig. S3** Viability of HepG2-NTCP cells after URI1 overexpression. Cell viability following URI1 overexpression was assessed by XTT assay at 2, 4, and 7 days after transfection. Data are presented as the mean ± SD of three independent experiments, with triangles indicating the mean of each experiment.
